# FORMATIVE FACTORS INFLUENCING SOCIAL CARE SERVICES DEMAND AMONG OLDER ADULTS: A FORTITUDE PERSPECTIVE

**DOI:** 10.1093/geroni/igae098.2452

**Published:** 2024-12-31

**Authors:** Hengyuan Zhang, Xuena Li

**Affiliations:** Xi’an Jiaotong University, Xi’an, Shaanxi, China (People’s Republic); Xi’an Jiaotong University, Xi’an, Shaanxi, China (People’s Republic)

## Abstract

**Background:**

There is a serious mismatch between the supply and demand of social care services for older adults in China. The imprecise identification of formative factors leads to challenges for service providers in targeting the appropriate groups.

**Methods:**

Adopting a fortitude perspective, a theoretical framework to analyze the formative factors of social care services demand was developed. Based on survey data from three cities and six counties, the impact of these formative factors was examined through binary logistic regression analysis.

**Results:**

The demand for social care services among older adults is significantly influenced by their physical, familial and resource fortitude, following an overflow pattern of “individual-family-market-government”. Conclusions: Demand for social care services emerges only when the challenges faced surpass the physical, familial, and resource fortitude of older adults. Overprovision to those with high fortitude and overlooking individuals with low fortitude results in a supply-demand imbalance.

